# Application of large-scale grid-connected solar photovoltaic system for voltage stability improvement of weak national grids

**DOI:** 10.1038/s41598-021-04300-w

**Published:** 2021-12-31

**Authors:** Bukola Babatunde Adetokun, Joseph Olorunfemi Ojo, Christopher Maina Muriithi

**Affiliations:** 1 Department of Electrical Engineering, Pan African University Institute for Basic Sciences, Technology and Innovation, Jomo Kenyatta University of Agriculture and Technology, Nairobi, Kenya; 2 Present Address: Department of Electrical and Electronics Engineering, Faculty of Engineering, Nile University of Nigeria, Abuja, Nigeria; 3 Department of Electrical and Computer Engineering, Tennessee Technological University, Cookeville, TN USA; 4 Department of Electrical Power Engineering, Durban University of Technology, Durban, South Africa; 5 Department of Electrical Engineering, Murang’a University of Technology, Murang’a, Kenya

**Keywords:** Electrical and electronic engineering, Energy grids and networks, Photovoltaics

## Abstract

This paper investigates the application of large-scale solar photovoltaic (SPV) system for voltage stability improvement of weak national grids. Large-scale SPV integration has been investigated on the Nigerian power system to enhance voltage stability and as a viable alternative to the aged shunt reactors currently being used in the Nigerian national grid to mitigate overvoltage issues in Northern Nigeria. Two scenarios of increasing SPV penetration level (PL) are investigated in this work, namely, centralized large-scale SPV at the critical bus and dispersed large-scale SPV across the weak buses. The voltage stability of the system is evaluated using the active power margin (APM) also called megawatt margin (MWM) derived from Active Power–Voltage (P–V) analysis, the reactive power margin (RPM) and the associated critical voltage–reactive power ratio (CVQR) index obtained from Reactive Power–Voltage (Q–V) analysis. All simulations are carried out in DIgSILENT PowerFactory software and result analyses done with MATLAB. The results show that with centralized SPV generation for the case study system, the highest bus voltage is able to fall within acceptable limits at 26.29% (1000 MW), while the dispersed SPV achieves this at 21.44% (800 MW). Also, the dispersed SPV scenario provides better voltage stability improvement for the system as indicated by the MWM, RPM and the CVQR index of the system. Therefore, this work provides a baseline insight on the potential application of large-scale SPV in weak grids such as the Nigerian case to address the voltage stability problems in the power system while utilizing the abundant solar resource to meet the increasing energy demand.

## Introduction

Renewable power generation is gaining prominence in the global energy market. This is mainly necessitated by the drive towards clean, sustainable energy in order to mitigate greenhouse emission effects on the climate and to minimize dependence on fossil fuel^1^. As an instance of global commitments, the European Union aims to phase out fossil-fuel based generations and to achieve a 100% renewable power generation by the year 2050^2^. Significant amount of US dollars are also being invested in the development of renewable energy systems in China, North America, India, Brazil and Japan^3^. In addition, considerable efforts are being made in Africa and Middle-East to utilize renewable energy sources for electrical power generation. Thus, there is significant increase in investments and development of renewable energy conversion systems globally^4–7^. Wind and solar are the leading renewable energy resources, which can be harnessed to generate large amount of electric power suitable for grid integration. Presently, more than 651GW of wind energy conversion systems are installed globally^8^. In addition, design and development of grid-connected solar PV (SPV) system is on the increase as the technology usage is shifting from the conventional small-scale rooftop to utility-scale grid-connected SPV. About 290GW of SPV has been installed by the end of 2016^9^.

Various studies have been carried out on large-scale SPV integration into the power grid. A review of important power system stability issues associated with large-scale SPV integration into power grid has been carried out in^2^. The authors in^10^ proposed a techno-economic approach to enhance SPV connectivity. In^11^, a generation and transmission expansion planning based on co-optimization model has been proposed in order to maximize SPV hosting capacity in power systems. In addition, a real-time model to predict the efficiency and output power of grid-tied SPV systems has been proposed in^12^. The authors in^13^ deals with the development of a non-linear control scheme and stability assessment for a single-stage grid-connected SPV. A stochastic approach to study the effects of SPV on statistically-generated low voltage distribution grids, which facilitates fast and efficient procedures for SPV impact assessment on similar networks has been proposed in^14^. The study in^15^ addresses modelling and stability issues in distributed grid-integrated SPV systems employing DC optimizer-based maximum power point tracking by proposing a matrix-based method to obtain an average model that reduces the computational burden. This model was then employed to assess the small signal stability of the system. Also, small signal stability analysis of grid-integrated SPV using a data-driven polynomial chaos expansion approach has been proposed in^16^.

Furthermore, the study carried out in^17^ indicates that SPV systems with improved controllers can improve dynamic reactive power response, thereby enhancing long-term voltage stability of the grid. The study also noted that large-scale grid-integrated SPV can have both beneficial and adverse effects on stressed power grids. In^18^, a review on large-scale SPV integration approach into weak power grids and the attendant influence on voltage stability has been discussed. The study employed particle swarm optimization algorithm to carry out a techno-economic analysis of the study system, considering three distinct SPV penetration level. Also, in^19^, a utility-scale, grid-friendly SPV system that incorporates advanced capabilities required to support grid stability and provide other ancillary services essential to the reliability of the grid has been described. The authors in^20^ investigated the impact of increased SPV penetration on small signal stability of large power system. Eigenvalue analysis was used to determine the critical modes of the system and the effect of SPV penetration level on the identified modes. The effect of utility-scale grid-tied SPV on the voltage stability of power system was also examined in^21^. The results of the study indicate that significant SPV penetration can enhance the system’s voltage profile and mitigate voltage instability.

In particular, performance analysis of large-scale solar power integration for both developed and developing countries and regions have been carried out in several studies. The performance of a large-scale solar-photovoltaic power plant in Northern part of Ghana has been assessed in^22^. Similar analysis was carried out for North-eastern Brazil^23^, Japan^24^, Iraq^25^, Poland^26^, China^27^ and India^28–32^. The prospects of grid-connected SPV in Kenya has been investigated in^33^ and the authors in^34^ examined the possibilities of grid-connected SPV in Hong-Kong. A study on the spatial matching of utility-scale grid-tied SPV with utility demand in Peninsular Malaysia was carried out in^35^. The effects of integrating solar microgrid system into Swedish power grid on climate change was also investigated in^36^. Also the effects of large SPV integration into Egypt’s power grid has been studied in^37^ and the study in^38^ assessed the impact of large-scale SPV and wind integration on the voltage stability of Jordan’s national grid.

Furthermore, a comparative study on the effect of distributed and centralized large-scale SPV on Ontario’s power system stability has been conducted in^39^. Eigenvalue, voltage stability and transient stability analyses were carried out for three different cases of SPV system integration into the system. The results show that distributed SPV provide better system stability than a single centralized solar farm. In^40^, a feasibility assessment regarding 100% RE grid in Japan was performed by investigating cases of future wind, solar, and tidal energy generation. The study indicates that incorporating energy storage into the system can enhance the system’s stability. Moreover, the impact of high SPV and wind penetration on the frequency response of South Australia’s power system has been studied in^41^. Also, the study in^42^ showed that high RE penetrations of greater than 80% is achievable in the Texas grid.

The possibilities, prospects and challenges of large-scale SPV generation in Nigeria have been investigated in some studies. The key barriers to solar energy implementation are discussed in^43^. The results of the analysis carried out in^44^ indicate that Nigeria’s transition to a sustainable and renewable power generation through utility-scale solar power generation can lessen global warming effects and diversification of energy sources can be achieved. A particular scenario of selected location in Northern Nigeria has been investigated for the feasibility of grid-connected SPV generation using an energy optimization software in^45^. The findings show that development of grid-connected SPV system in Northeastern part of Nigeria is economically viable. Moreover, a generation planning incorporating SPV systems in the Nigerian grid has been presented in^46^ and the prospects of hybrid SPV-diesel energy systems in some parts of Northern Nigeria has been analysed in^47^. The study in^48^ focussed on the effects of high SPV penetration on the West African power pool using a multi-region economic dispatch model Furthermore, in^49^, a solar energy roadmap for clean and sustainable energy technology investment in the abundant solar energy of Northeastern Nigeria has been presented. The techno-economic and environmental sustainability of installing grid-integrated SPV in the region was validated using RETScreen Expert software and climatic data obtained from National Aeronautics and Space Administration. The results show the viability of all the selected locations in Northern Nigeria for large-scale SPV generation. These works thus show that large-scale SPV is a viable and sustainable energy option for the Nigerian electricity sector.

However, the paucity of studies on the use of large-scale SPV integration in weak grids of a developing country and the attendant voltage stability improvement benefits has necessitated this present study. Therefore, the application of utility-scale grid-integrated SPV system to improve the voltage stability of a weak national grid has been carried out in this work. The Nigerian power system has been used as a case study in this work. In addition, the voltage stability study in this work has been compared with similar aspects of studies carried out in other parts of the world. This study investigates the impact of increasing SPV power penetration on the active and reactive power margins of the system to determine the optimal and most appropriate penetration level for the present Nigerian system. Furthermore, the CVQR obtained from Q–V curves has been utilised in this work to evaluate the tendency of power system towards voltage instability as the SPV penetration level increases. In order to validate the accuracy of the CVQR, its performance has been compared with the tangent vector index obtained from P–V analysis and the reactive power margin derived from Q–V curve. This study also highlights the potential application of large-scale, grid-integrated SPV systems located in Northern Nigeria as a viable alternative to the aged shunt reactors presently in use to mitigate overvoltage occurrences in the Northern region. Practical considerations regarding the reactive power limits of conventional generators and the SPV system have been taken into account in this study. In this work, the SPV power penetration level is taken as the proportion of the total real power produced by SPV to the overall real power dispatched from all the generators within the system. Similar definition was utilised in^20,50^. The value of the total active power generated, which is the denominator, is determined by the load flow calculations and it varies for each case of RE penetration because the active power losses are different at each penetration level.

The main contributions of this work are highlighted as follows:

- This work has investigated the application of large-scale grid-connected SPV to enhance the voltage stability of weak power grids with a particular case study system.
- Scenarios of increasing large-scale SPV penetration level for centralized and dispersed locations are studied and compared. This provides a more comprehensive insight into the application of large-scale SPV than studies that concentrate only on centralized SPV application.
- The accuracy of the derived Q–V based CVQR, which measures the voltage instability tendency of power grids with increasing SPV penetration level has been validated by comparing its performance with tangent vector index and the system’s reactive power margin. In addition, this study utilises active power margin derived from P–V analysis and reactive power margin obtained from Q–V analysis to assess the impact of increasing large-scale SPV integration on the voltage stability of a weak power grid. Therefore, this work presents a more robust study by considering both P–V and Q–V behaviour of a weak power system with increasing SPV integration.
- The uniqueness of this work is in the application of comprehensive P–V and Q–V based indices to analyze the potentials of large scale solar photovoltaic systems for voltage stability improvement of national power grids that are prone to voltage instability problems. The analysis in this work shows that large-scale SPV can be a viable alternative for reactive power absorbing devices such as shunt reactors for voltage stability improvement while generating the required active power needs of connected loads.
- This study has been compared with studies on other national grids and the analysis carried out in this work can be applied to other weak national grids, where there is sufficient solar energy resource for power generation.

The rest of this paper is arranged as follows: “Current Status of the Nigerian National Grid and the Solar Energy Potentials” section presents an overview of the 330 kV Nigerian power grid. In “Current Status of the Nigerian National Grid and the Solar Energy Potentials” and “P–V and Q–V curves analyses” sections briefly present how the CVQR and tangent vector are derived respectively. In “Voltage stability analysis” section, the results for various scenarios are presented and discussed. In “Comparison of this study with other regions” section provides the comparison of this study with studies particular to other parts of the world and the conclusion is provided in “Conclusion” section.

## Current status of the Nigerian national grid and the solar energy potentials

This section presents the background information on the current status and issues of the 330 kV Nigerian National grid and the solar energy potentials of the country.

### Status of the Nigerian 330 kV national grid

**Figure 1:**
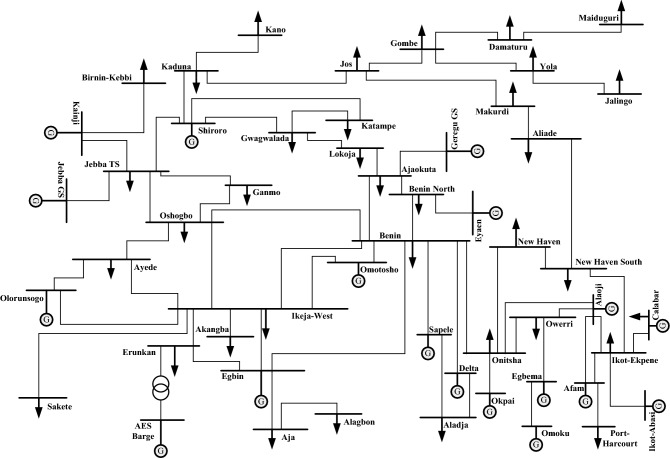
One-line diagram of 52-bus, 330 kV Nigerian power grid.

The current Nigerian national grid consists of a 330 kV, 52-bus system with 18 generator buses and 65 transmission lines. A representative one-line diagram of the system is depicted in Fig. 1. Detailed parameters of the system have been provided in^51^. The data obtained from the Transmission Company of Nigeria (TCN) and the Fichtner’s report on transmission expansion plan contained in^52^ indicate that the gross total installed power generation capacity is about 13,300 MW with a net capacity of 11,800 MW. However, based on the TCN statistics, only 5900 MW net capacity has been available. Furthermore, due to various outages, the actual peak generation is rarely above 4000 MW. As at 2019, the operational generation capacity is about 3810^53^. A practical total active power schedule of 3702 MW has been used for the analyses in this work. This corresponds to the current average daily schedule.

The problems of Nigerian Power Grid include old, inefficient power grid equipment, poor loadability and insufficient generation particularly at the Northern part of the country. Because of the long 330 kV transmission lines built to supply the Northern areas, overvoltage issues occur at the Northern buses^54^. Thus, shunt reactors are employed in selected Northern buses to absorb the excess reactive power, which is the cause of the overvoltage issues^52^.

Furthermore, the shortage of power supply inevitably results to load shedding. This implies that only some portion of the total load demand can be met by the available power dispatched. Therefore, considering the effect of load shedding due to limited energy generation, a practical average load demand of 3658 MW has been chosen for the analyses in this work, which is about 36% of the overall active power demand.

At present, power generation is mainly in the South region, which is close to the oil and gas supplies. Considering generation expansion planning, there is a need for new generating stations to be developed in the North also. However, development of large-scale gas-fired generating stations in the North may be extremely challenging due to the very long distance of these areas to the oil and gas fields located in the Niger-Delta region of Southern Nigeria. Since the solar energy potentials in the North are yet to be seriously harnessed in considerably large scale, a proper generation expansion plan must include solar energy resource, which is abundantly available in the North.

### Solar energy potentials

**Figure 2:**
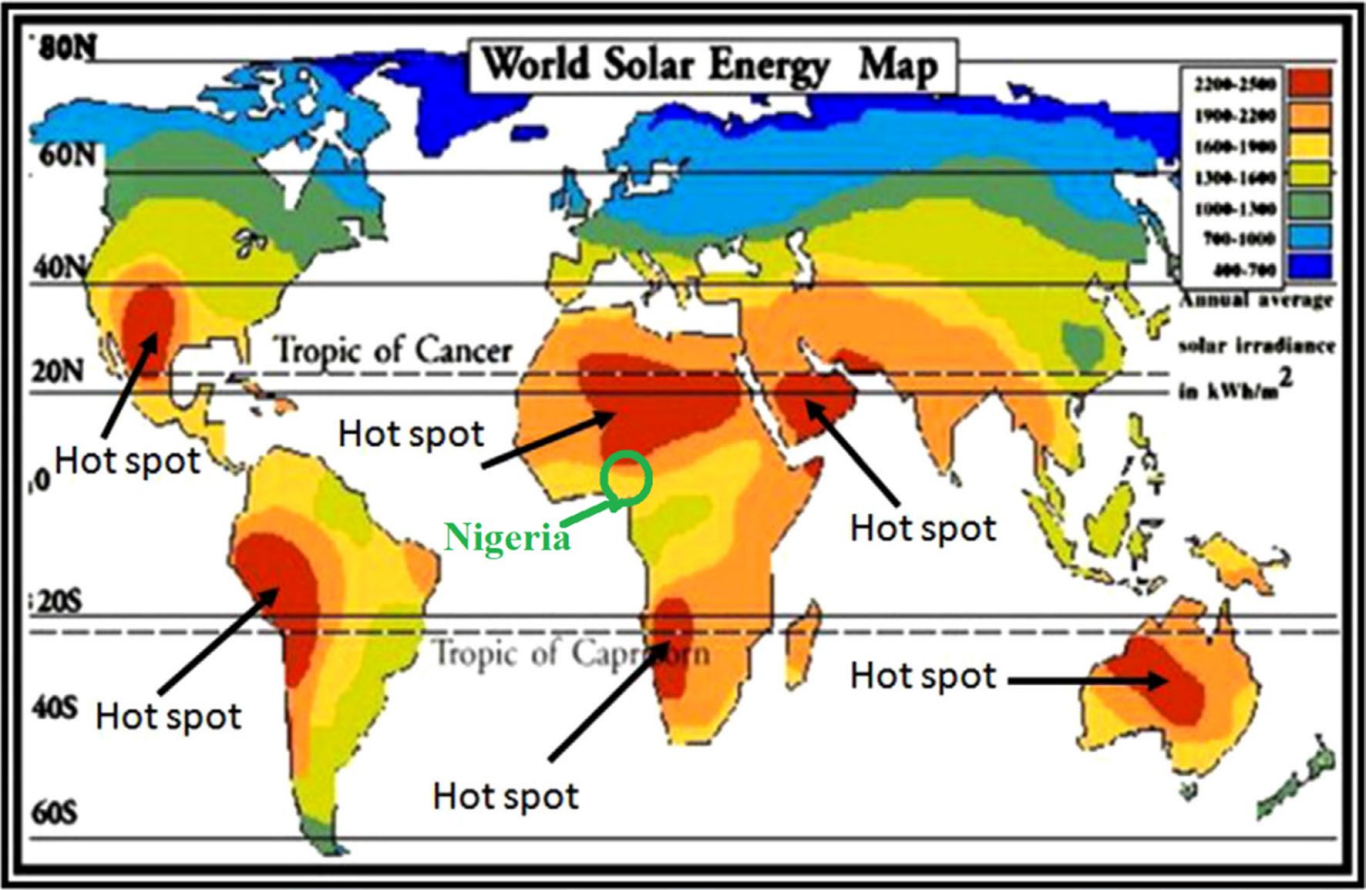
World solar energy map^55^.

Approximately 1.8 × 10^11^ MW of solar power is received on the earth surface each moment^2^. Thus, solar power generation is a promising renewable energy alternative in the world in general and in Africa in particular. The world solar energy map is shown in Fig. 2^55^. The map shows that Africa is literally lit with abundant solar irradiation and actually has the highest concentration of solar energy in the world. Thus, Africa has enormous potentials for solar power generation, which are largely untapped. The location of Nigeria, which is our case study in this work is indicated on the map. Nigeria is located in West Africa and within latitude 4.32°N and 14°N and longitude 2.72°E and 14.64°E with a total area of 923,768 km^2^. Nigeria is thus shown to have abundant solar irradiation ranging from a minimum of 1600 kWh/m^2^/year in the southern parts to 2500 kWh/m^2^/year in the Northern parts.

The present Nigerian economy is pre-eminently driven by fossil-fuel. This makes the energy sector to be largely oil-dependent and Nigeria has not been able to significantly harness the abundant solar resource for utility-scale electricity generation. Insufficient generation, frequent electric power blackouts and grid collapse are some of the bane of the Nigerian power sector. Also, there are numerous forced outages due to insufficient gas supply for the gas-fired thermal stations. The incessant power shortages has led to proliferation of petrol and diesel-fired generating sets for domestic, commercial and industrial uses.

Therefore, deliberate policy implementations and focus on renewable energy development is of utmost importance in meeting the energy needs of the people and in limiting reliance on fossil fuel^46^.

## P–V and Q–V curves analyses

The static analysis has been carried out using P–V curve and Q–V curve analyses. The concepts of P–V and Q–V analyses are well-established. Thus, we have presented how these analyses are utilised to determine the weakest buses in the power system network.

### Active power margin and tangent vector (TV) analysis

The active power margin (APM), also called the megawatt margin (MWM) of the system can be derived from the maximum scalable demand derived from P–V curve analysis and the base case active power demand. The P–V analysis from which the MWM of the system is derived provides an insight on how increase in load demand affects the system’s voltage stability. The megawatt margin indicates the extent to which the system can respond to increase in energy demand. Therefore, P–V analysis provides framework for considering changes in load demand and loadability of the system.

**Figure 3:**
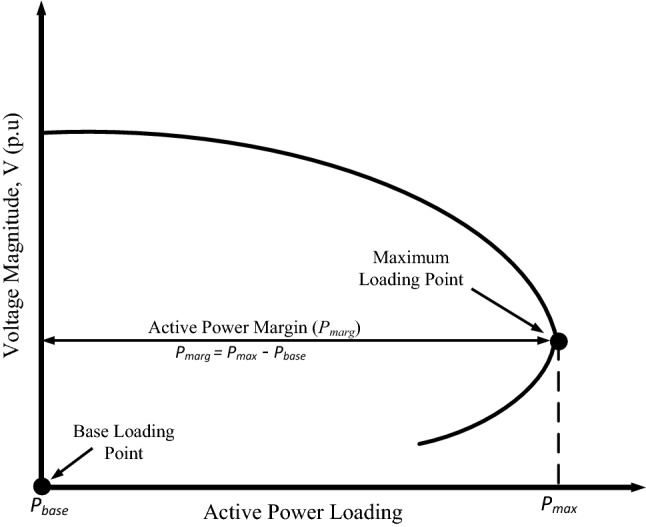
P–V curve showing the active power margin.

Figure 3 illustrates the P–V curve, indicating the loadability of the system as measured by the APM. The APM denoted as *P*_*marg*_, is the difference between the maximum active power demand (*P*_*max*_) and the base case demand (*P*_*base*_). This can be expressed as:

1 $$P_{marg.} = P_{max} - P_{base}$$

The loading factor, *λ* is a multiplication factor, which defines the load increment. Thus, the active power loading is a function of the loading factor and is defined as^1^:

2 $$P\left( \lambda \right) = P_{base} \left( {1 + \lambda } \right)$$

When *λ* = 0, this indicates base load since *P(λ)* will be equal to *P*_*base*_. Also, *λ* = *λ*_*max*_ indicates the maximum loading, *P*_*max*_.

The critical bus (CB) can be identified using P–V curve analysis by computing the tangent vector for each bus. The tangent vector elements are the ratios of differential change in voltage (*dV*) to differential change in load (*dP*) for all the buses. The critical bus has the largest tangent value (*dV/dP*) in the last converging iteration-step (*L*th iteration) of the continuation power flow process. Therefore, for any *n*-bus system, the critical bus is defined as:

3 $$CB = \max \left\{ {\left| {\frac{{dV_{1} }}{dP}} \right|,\left| {\frac{{dV_{2} }}{dP}} \right|\left| {\frac{{dV_{3} }}{dP}} \right|,...\left| {\frac{{dV_{n} }}{dP}} \right|} \right\}^{L}$$

### Critical voltage-reactive power ratio (CVQR) and reactive power margin (RPM)

**Figure 4:**
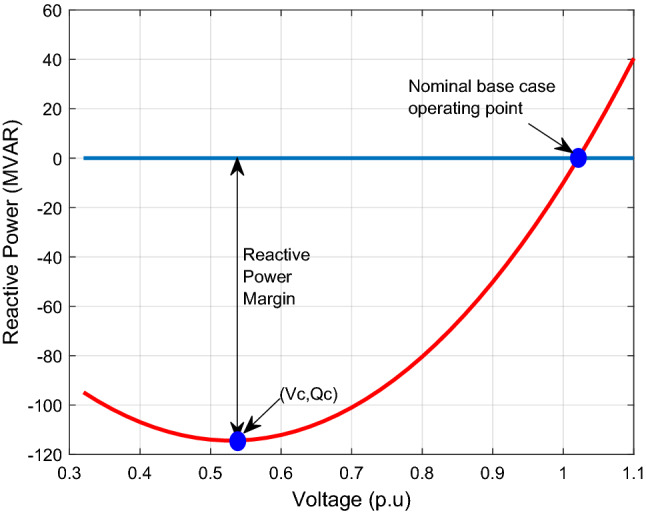
QV curve analysis showing RPM and CVQR.

The CVQR and RPM can be obtained from Q–V analysis for all buses as illustrated in Fig. 4. The reactive power margin is a measure of the largest reactive load that can be accommodated by a bus before voltage collapse occurs. The critical voltage is that voltage at which the maximum reactive power is attained. The CVQR is therefore the ratio of the critical voltage (*V*_*c*_) to the maximum reactive power (*Q*_*c*_). The *Q*_*c*_ is negative for normal operating conditions and positive when voltage collapse occurs. Therefore, CVQR is negative for normal operating conditions, however, the more negative the CVQR of a given bus is, the more unstable the bus becomes. A positive CVQR indicates that voltage collapse has occurred. The critical bus (CB) can be determined using this approach and information regarding the voltage stability condition of the system can be obtained. Therefore, in identifying the critical bus of any *n*-bus system using CVQR, the critical bus is determined as:

4 $$CB_{CVQR} = \min \left\{ {\frac{{V_{c1} }}{{Q_{c1} }},\frac{{V_{c2} }}{{Q_{c2} }},\frac{{V_{c3} }}{{Q_{c3} }}, \ldots \frac{{V_{cn} }}{{Q_{cn} }}} \right\}$$

Equation (3) is applicable when all the CVQR values are negative. If the CVQR associated with any bus is positive (CVQR > 0), then voltage collapse has occurred.

These QV-based indices are applied in the static analysis to rank the power system buses and their performance compared to the P–V curve tangent vector approach.

## Voltage stability analysis

Three cases are investigated in this section. The results of these cases are presented and discussed here. The reactive power limits of conventional generators and the reactive power capability of large-scale solar photovoltaic (SPV) system have been put into consideration in this study. For the continuation power flow study, the load increase is uniformly distributed for all the load buses and the loads are modelled as constant PQ load. The values of the active and reactive power of the load utilized in this work are specified in Table 1.

**Table 1:** Load data of the 52-bus 330 kV Nigerian power grid.

Load bus	P (MW)	Q (MVAR)	Load bus	P (MW)	Q (MVAR)	Load bus	P (MW)	Q (MVAR)
Bus 2	188	91	Bus 19	113	55	Bus 36	60	30
Bus 3	321	155	Bus 20	71	34	Bus 38	12	6
Bus 4	297	144	Bus 21	189	62	Bus 39	60	30
Bus 5	91	44	Bus 22	60	29	Bus 40	15	7
Bus 6	170	82	Bus 24	102	48	Bus 41	10	6
Bus 9	148	72	Bus 25	167	78	Bus 46	50	20
Bus 10	170	82	Bus 26	140	64	Bus 48	18	8
Bus 12	260	125	Bus 31	139	65	Bus 49	16	7
Bus 14	71	34	Bus 32	105	51	Bus 50	10	4
Bus 16	157	76	Bus 33	60	40	Bus 51	14	6
Bus 17	173	84	Bus 34	50	30	Bus 52	17	8
Bus 18	89	43	Bus 35	45	25	Total	3658	1745

### Scenario 1: without shunt reactors

**Figure 5:**
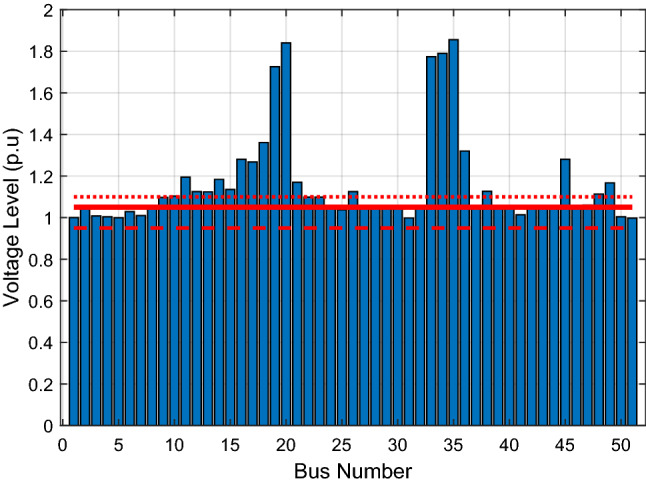
Bus voltage profile of the Nigerian 52-bus system without shunt reactors.

The voltage profile for each bus when no shunt reactor or any reactive power compensation device is utilised is presented in this section. The voltage profile for this base case scenario is shown in Fig. 5. The dashed, solid, and dotted red lines indicate the 0.95 pu, 1.05 p.u and 1.1 p.u voltage levels respectively. The figure reveals that overvoltage condition occurs in some buses, particularly in the Northern areas. This is due to the boost in the reactive power along the considerably long transmission lines connecting the central region to the Northern areas and the lightly loaded conditions of the Northern buses. This explains the reason for the use of shunt reactors in the Nigerian system. Shunt reactors absorb excess reactive power in order to keep the bus voltages at an acceptable level. With the highest voltage reaching as high as 1.86 p.u, the grid is forced to shut down. There are reported cases of deliberate grid collapse initiated by the National Control Centre, Oshogbo, because of sudden, dangerous overvoltage occurrence. Thus, the present Nigerian grid cannot safely operate without the application of any reactive power absorbing device, such as shunt reactors.

### Scenario 2: shunt reactors applied in five locations

**Figure 6:**
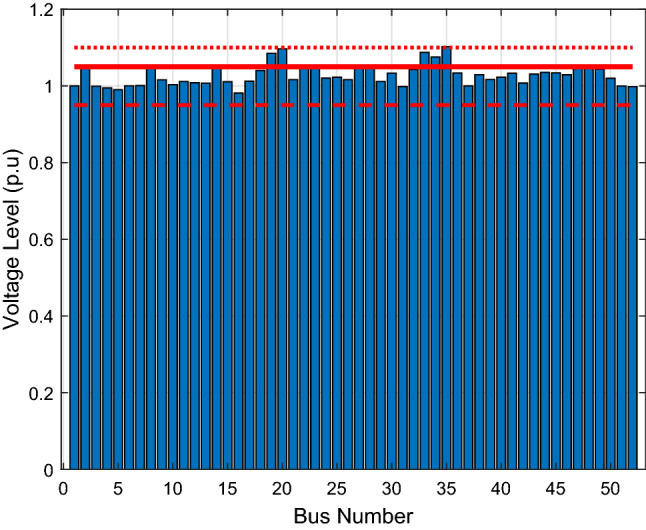
Bus voltage profile of the Nigerian 52-bus system with shunt reactors at five locations.

In this second scenario, we consider the effects of shunt reactors on the system’s voltage stability. This is the present case of the Nigerian grid. Shunt reactors are used to absorb the excessive reactive power, which produces overvoltage issues in the Northern buses. Figure 6 shows the bus voltage as determined from the load flow study of the system when shunt reactors are employed in five locations (Kaduna-75MVAR, Kano-75MVAR, Gombe-100MVAR, Yola-100MVAR and Maiduguri-75MVAR). It can be observed that the overvoltage issue in the base case scenario is significantly mitigated. However, the voltage levels at buses 19 (Gombe), 20 (Yola), 33 (Damaturu), 34 (Maiduguri) and 35 (Jalingo) still exceed 1.05 p.u, but not much beyond 1.10 p.u. This is still consistent with the overvoltage occurrence in Northern Nigeria. Thus, with the use of shunt reactors at Kano, Yola, Kaduna, Maiduguri, and Gombe, only one of the bus voltage levels slightly exceed 1.10 p.u as depicted in the figure. The voltage level at Jalingo, which is 1.1023 p.u is the highest. However, the performance of the shunt reactors is not fully satisfactory, coupled with the fact that the shunt reactors presently in use in Nigeria are aged and subject to frequent failures.

**Figure 7:**
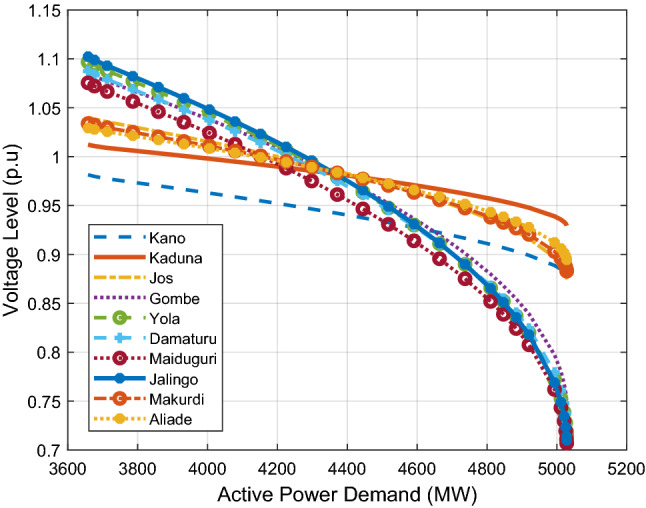
P–V Curves for the critical northern buses.

Furthermore, P–V and Q–V curves analyses are carried out to determine the critical loading limit and the reactive power margin of the system respectively. Figure 7 depicts the P–V curve for the most critical buses, which are in the Northern areas. The figure shows that with a base case total active load demand of 3658 MW, the total critical active power demand is 5028 MW and the tangent vector analysis of the P–V curves shows that Jalingo bus (bus 35) is the weakest bus in the system, followed by Yola, Maiduguri, Damaturu, Gombe, Jos, Makurdi, Aliade, Kano and Kaduna in that order.

The Q–V curve results also indicate that Jalingo has the smallest RPM (139.57MVAR) and the most negative CVQR of − 0.459. This shows that Jalingo bus is the weakest bus in the expanded 52-bus Nigerian 330 kV Power Grid. This result is similar to the weakest bus identification carried out in^56^ using eigenvalue method, where the Jalingo bus was also identified as the critical bus in the 330 kV, 52-bus Nigerian power grid. Table 2 shows the first ten weakest buses according to the tangent vector, reactive power margin and CVQR index rankings. This table gives a baseline comparison of the derived indices used in this work and the significant agreement in the bus rankings provides a basic validation for these methods. These results invariably show that buses in the northern parts are the weakest in the Nigerian power grid in terms of active and reactive power loadability margins. This is because these buses are largely distant from the southern buses where generating stations are concentrated.

**Table 2:** Bus Ranking of Nigerian 330 kV power grid: comparison of P–V and Q–V-based indices.

Ranks	Bus	TV	Bus	RPM	Bus	CVQR
1	Jalingo	0.7266	Jalingo	139.57	Jalingo	− 0.4586
2	Yola	0.7065	Maiduguri	152.83	Yola	− 0.4286
3	Maiduguri	0.6755	Yola	153.99	Maiduguri	− 0.4122
4	Damaturu	0.6636	Damaturu	172.16	Damaturu	− 0.3892
5	Gombe	0.6171	Gombe	201.7	Gombe	− 0.3471
6	Jos	0.2687	Kano	403.3	Jos	− 0.1439
7	Makurdi	0.2407	Birnin-Kebbi	451.23	Kano	− 0.1438
8	Aliade	0.2114	Jos	528.26	Makurdi	− 0.1296
9	Kano	0.1207	Makurdi	578.78	Birnin-Kebbi	− 0.1285
10	Kaduna	0.1104	Aliade	648.84	Aliade	− 0.1140

### Scenario 3: large-scale solar PV integration in the northern region

In this scenario, we investigate the possibility of utilising large-scale solar PV integration to enhance the voltage stability of the Nigerian grid while meeting the rising energy demand of the country. Two cases are considered here. In the first case, large-scale solar PV generation is located at Jalingo, since it has been determined as the weakest bus of the system, and the state where Jalingo is located has been reported to be suitable for solar power generation. In the second case, solar PV is distributed throughout selected buses in the Northern region, where there is abundant solar resource. The solar PV is modelled as a generator (PV) bus in this analysis. The reactive power limits of conventional generators and the reactive power capability of large-scale solar photovoltaic (SPV) system have been put into consideration in this study. Details of the reactive power control and capability characteristics of both the conventional synchronous generator and the SPV system have already been presented in^17^.

#### Solar PV located and centralized at Jalingo

The impact of increasing Solar PV penetration at the Jalingo bus on the voltage stability of the system has been carried out in this section. The Solar PV integration is examined for penetration levels ranging from 100 MW (2.65% PL) to 1000 MW (26.29% PL). The impact of increasing SPV penetration on the bus voltage profile is carried out with load flow studies. In addition, the effects of the increasing PL on the voltage stability margins of the SPV-connected grid are assessed with P–V and Q–V methods. The P–V analysis gives the total active power margin of the system and the Q–V study provide the reactive power margin and the CVQR index of the system for each investigated penetration level.

**Figure 8:**
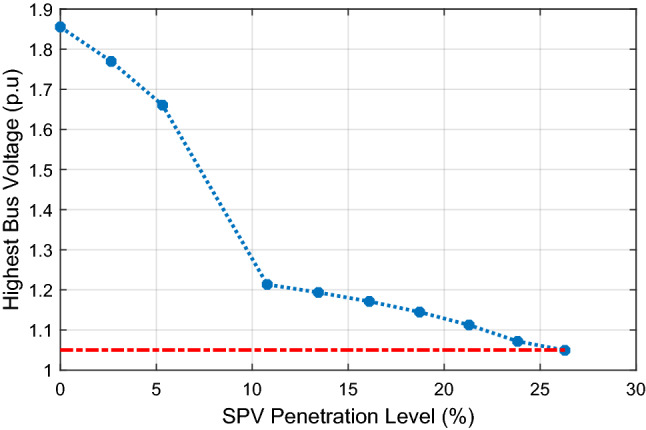
Impact of increasing SPV penetration on highest bus voltage.

The impact of increasing SPV PL on the highest bus voltage within the system is illustrated in Fig. 8. The figure indicates that the highest bus voltage decreases as the SPV PL increases and falls within 1.0 ± 0.05 p.u at about 26.29% PL (1000 MW). This implies that the optimal SPV PL at Jalingo bus that will not lead to voltage limit violation at any bus is 1000 MW. Further increase in SPV PL at Jalingo bus results in low voltage condition (bus voltage less than 0.95 p.u) at Jos, Gombe, Yola, Damaturu and Makurdi. Since low bus voltage is undesirable, the SPV PL at Jalingo should not exceed 1000 MW.

**Figure 9:**
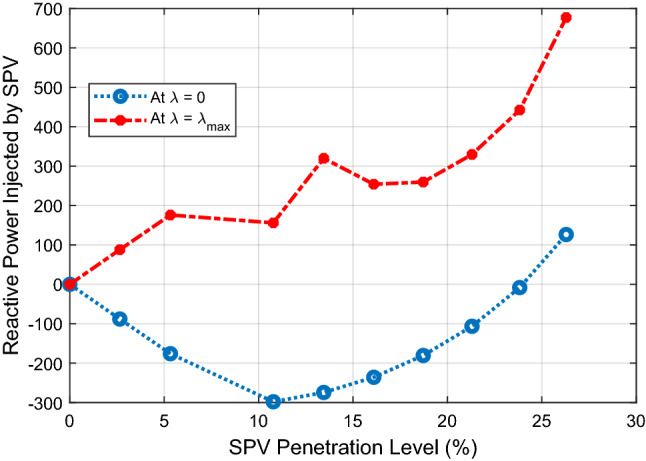
Reactive power injected by the SPV.

The reactive power absorbed/injected by the SPV at base case loading condition (*λ* = 0) and at maximum loading condition (*λ* = *λ*_*max*_) is depicted in Fig. 9. The figure shows that the SPV absorbs reactive power from the system at base case loading in order to regulate the bus voltages. The reactive power absorbed by the SPV is highest at 10.78% PL. After this point, the reactive power absorbed continues to decrease with increasing SPV PL in order to ensure that the system does not suffer voltage collapse due to declining reactive power in the system. At 26.29% PL, the SPV injects about 126.4MVAR into the system in order to sustain the voltage stability of the grid at this high penetration level. During maximum loading of the system as indicated by *λ* = *λ*_*max*_ line in Fig. 9, the SPV injects reactive power into the system at each SPV penetration level so as to give voltage support to the system.

**Figure 10:**
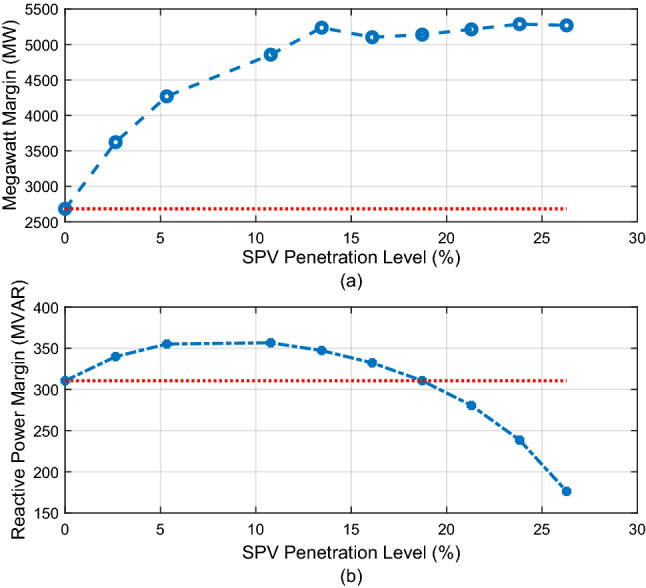
Variation of megawatt margin and reactive power margin with SPV PL.

The megawatt margin and the lowest reactive power margin of the system with respect to increasing SPV PL are shown in Fig. 10a and b. As illustrated in Fig. 10a, the megawatt margin of the system continues to improve significantly as the SPV penetration level increases. Figure 10b indicates that the minimum reactive power margin of the system initially improves with increasing SPV PL and peaks at 10.78% SPV PL. This corresponds to the point at which the reactive power absorbed by the SPV is highest as depicted in Fig. 9. Thereafter, the RPM begins to decline and it returns to its original base case value at 18.72% (700 MW). The red dotted line in each figure indicates the original base case value. The RPM falls to a value of 176.3MVAR at the last investigated SPV PL of 26.29%.

**Figure 11:**
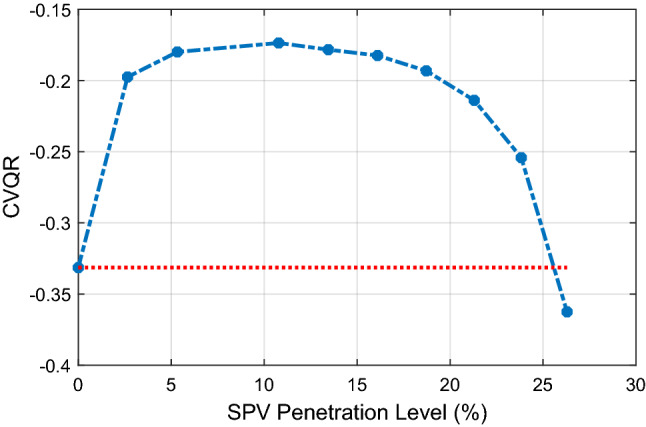
Variation of CVQR index with increasing SPV PL.

The corresponding CVQR of the system associated with the minimum RPM is shown in Fig. 11. The figure shows that the voltage stability of the system is initially improved as indicated by CVQR value becoming less negative. However, the CVQR begins to become more negative after 10.78% SPV PL, and returns to its original value at about 25.6% SPV PL. It can be observed from Figs. 10 and 11 that although the loadability of the system is increasingly enhanced with increasing SPV PL as indicated by the MWM of the system, the RPM and the CVQR of the system declines at higher SPV PL, thereby showing that the system tends toward voltage instability at higher SPV PL.

#### Solar PV located at weakest buses in the northern region

In this case, the SPV is dispersed across the weak Northern buses. SPVs are located in Jalingo, Maiduguri, Yola, Gombe, Damaturu, Kano, Jos, Kaduna and Birnin-Kebbi in an incremental manner. The Solar PV integration ranges from 100 MW (2.65% PL) to 1800 MW (46.81% PL) for this case.

**Figure 12:**
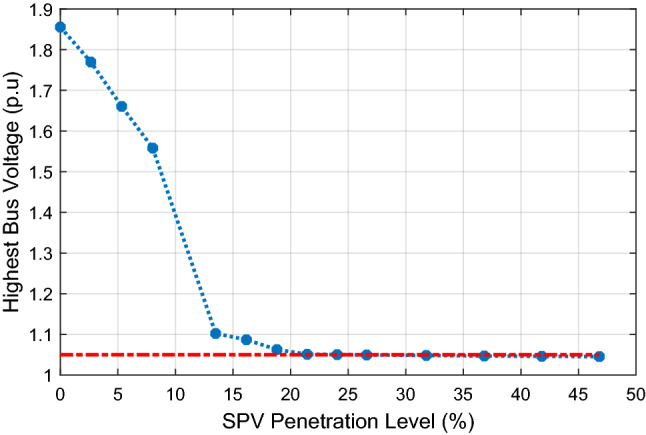
Impact of increasing SPV penetration on highest bus voltage with SPV located at selected Northern buses.

Figure 12 shows the variation of the highest bus voltage with respect to the SPV PL. The figure depicts that the highest bus voltage decreases as the SPV PL increases. The bus voltage criterion of 1.0 ± 0.05 p.u is achieved at about 21.44% PL (about 800 MW). Thus, with 200 MW SPV integration at Jalingo, Maiduguri, Yola and Gombe each, a satisfactory voltage profile can be attained, with additional advantages as compared to the use of shunt reactors. As observed from Fig. 12, SPV PL of 13.49% (500 MW) at three locations will ensure that no bus voltage exceeds 1.102 p.u, which is the maximum performance obtained from the use of shunt reactors at five locations as discussed in “Scenario 2: shunt reactors applied in five locations” section.

**Figure 13:**
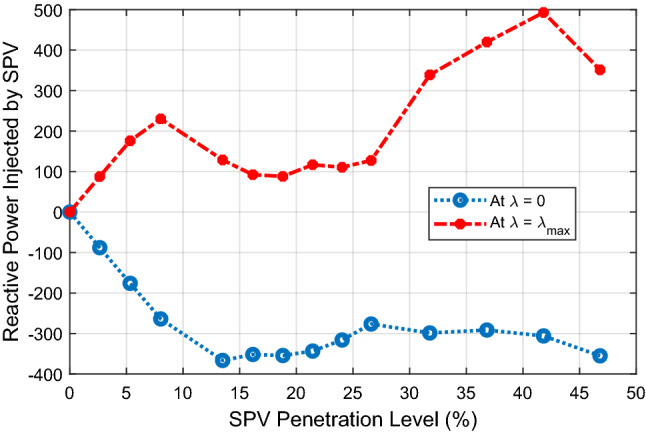
Reactive power absorbed/injected by the SPVs.

Figure 13 shows the variation of reactive power injected/absorbed by the SPVs as the PL increases. The figure shows that the SPV absorbs reactive power at nominal base case loading (*λ* = 0) and injects reactive power at maximum loading point (*λ* = *λ*_*max*_) for all SPV PLs.

**Figure 14:**
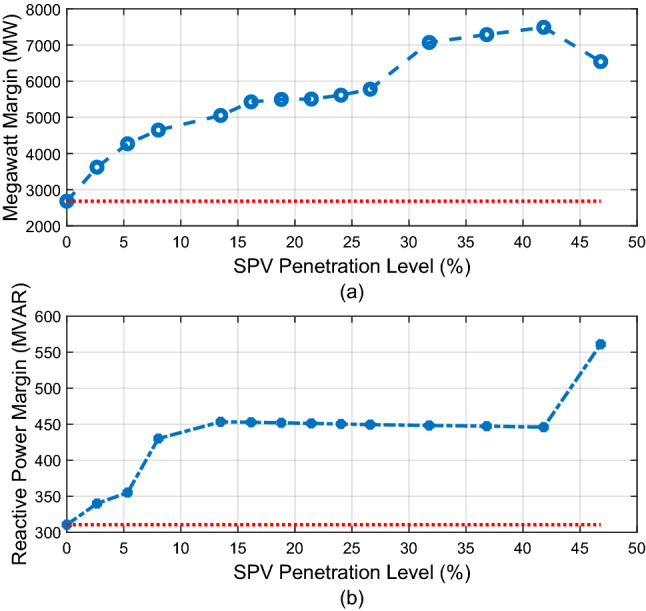
Variation of megawatt margin and reactive power margin with SPV PL.

**Figure 15:**
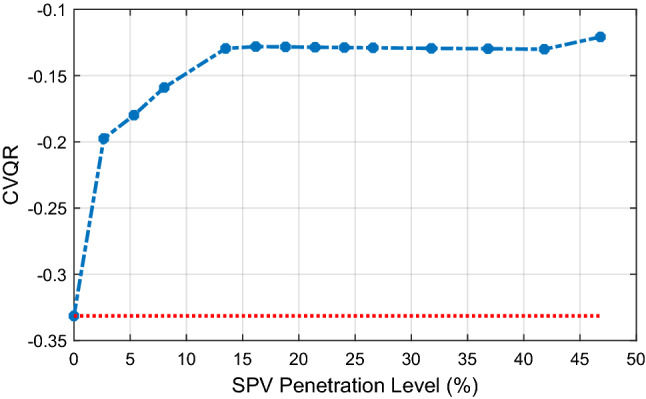
Variation of CVQR index with increasing SPV PL.

In Fig. 14a and b, the MWM and the minimum RPM of the system with respect to increasing SPV PL are illustrated. The figure shows that both the MWM and the RPM improves with increasing SPV PL. This is an additional benefit of employing large-scale SPV in various locations of the Northern region. Moreover, for this case, the corresponding CVQR of the system associated with the minimum RPM shown in Fig. 15 indicates that the voltage stability of the system is significantly improved as the SPV PL increases. Thus, this distributed SPV case offers a better voltage stability improvement than when SPV is located and lumped only at the Jalingo bus.

## Comparison of this study with other regions

This section provides a comparison of this work with voltage stability analysis of different national grids with large-scale SPV integration. Table 3 shows the comparison based on six aspects of each study case. The three regions considered are Egypt in North Africa, Jordan in Middle-East and Ontario (Canada) in North America. The comparison indicates that there are considerable efforts across different regions of the world to integrate utility-scale SPV systems and that dispersed large-scale SPV systems can improve static voltage stability of such national grids.

**Table 3:** Comparison of this present study with studies in other regions.

	This present study	Egypt region^37^	Jordan region^38^	Ontario (Canada) region^39^
Renewable energy system studied	Centralised and distributed grid-connected SPV	Distributed grid-connected SPV	Distributed grid-connected SPV and DFIG wind system	Centralised and distributed grid-connected SPV
Grid representation	330 kV, 52-bus system	426-bus, 66 kV, 132 kV, 220 kV and 500 kV networks	56-bus, 132 kV and 400 kV system	2975-bus system with voltage levels ranging from 4 to 500 kV
Load demand served	Between 3000 and 4000 MW for a typical day	Not available from the study	Between 1600 and 3400 MW	Peak summer demand of 25GW
Methodology	Highest bus voltage sensitivity, PV and QV based indices (APM, RPM and the associated CVQR)	P–V and Q–V curves	Variation of bus voltage level with active power generated by RE systems was used as parameter for the voltage stability study	Loadability margin of the system was used as a parameter for the voltage stability study
Simulation tools used	DIgSILENT PowerFactory and MATLAB	DIgSILENT PowerFactory	DIgSILENT PowerFactory	DSATools
Significant results	The optimal SPV penetration level that brings voltage level within acceptable limits is 1000 MW for centralized SPV generation and 800 MW for dispersed SPV generation. The dispersed SPV scenario provides better voltage stability improvement for the system as indicated by the MWM, RPM and the CVQR index of the system	Maximum Penetration Levels of up to 3000 MW was investigated. The results obtained from the P–V curve indicates that SPV PL at different locations was enhanced by voltage control mode of operation of the SPVs	Maximum Penetration Level of up to 10% of annual peak demand was determined based on the transmission line constraints. The maximum amount of RE integration can be significantly improved and the voltage profile enhanced when the RE plants are operated in voltage control mode	Penetration Levels of up to 1960 MW was investigated. The results of the voltage stability indicates that for the distributed SPV case, there is significant improvement in system loadability as the SPV PL increases, while the system loadability is nearly constant for the centralized SPV cases

## Conclusion

This work has investigated the potential application of large-scale solar photovoltaic system as a viable alternative to the aged shunt reactors currently being used in the Nigerian grid to mitigate overvoltage occurrences. In addition, the work shows that large-scale solar photovoltaic system is able to enhance the voltage stability margin of the system in terms of both megawatt and reactive power margins. The analysis shows that dispersed large-scale solar photovoltaic system across Northern Nigeria has better performance than centralized solar photovoltaic system at the critical bus, which is the Jalingo bus. With 500 MW of dispersed large-scale solar photovoltaic system, the highest voltage is brought to about 1.102 p.u which is the same performance obtained for the use of shunt reactors. However, increasing the penetration level of solar photovoltaic system to 800 MW brings all the bus voltages within recommended limits of 1.0 ± 0.05 p.u, while significantly improving the voltage stability of the system as shown by the critical voltage-reactive power ratio index. Therefore, this work has shown that employment of the vast solar resources in Northern Nigeria must be seriously considered in order to satisfactorily address voltage stability issues in the Nigerian system and to meet the increasing electrical power and energy demand of the country. The subject of energy storage modelling and analysis and its effects on voltage stability can be investigated in future studies. In addition, reliability study and transient analysis of grid-connected large-scale solar photovoltaic are worthwhile areas for further investigations.

## References

[1] Adetokun BB, Muriithi CM, Ojo JO (2020). Voltage stability assessment and enhancement of power grid with increasing wind energy penetration. Int. J. Electr. Power Energy Syst..

[2] Shah R, Mithulananthan N, Bansal RC, Ramachandaramurthy VK (2015). A review of key power system stability challenges for large-scale PV integration. Renew. Sustain. Energy Rev..

[3] Sharif A, Raza SA, Ozturk I, Afshan S (2019). The dynamic relationship of renewable and nonrenewable energy consumption with carbon emission: A global study with the application of heterogeneous panel estimations. Renew. Energy.

[4] Ayodele TR, Ogunjuyigbe ASO, Adetokun BB (2017). Optimal capacitance selection for a wind-driven self-excited reluctance generator under varying wind speed and load conditions. Appl. Energy.

[5] Ogunjuyigbe ASO, Ayodele TR, Adetokun BB (2017). Steady state analysis of wind-driven self-excited reluctance generator for isolated applications. Renew. Energy.

[6] Adetokun, B. B., Muriithi, C. M. & Ojo, J. O. Voltage stability analysis and improvement of power system with increased SCIG-based wind system integration. In *IEEE PES/IAS PowerAfrica*, vol. 2020, 1–5 (2020).

[7] Adetokun BB, Muriithi CM (2021). Application and control of flexible alternating current transmission system devices for voltage stability enhancement of renewable-integrated power grid: A comprehensive review. Heliyon.

[8] GWEC. *Global Wind Report 2019*. Available: https://gwec.net/global-wind-report-2019/ (Global Wind Energy Council, 2020).

[9] Nwaigwe KN, Mutabilwa P, Dintwa E (2019). An overview of solar power (PV systems) integration into electricity grids. Mater. Sci. Energy Technol..

[10] Othman MM, Ahmed HMA, Ahmed MH, Salama MMA (2020). A techno-economic approach for increasing the connectivity of photovoltaic distributed generators. IEEE Trans. Sustain. Energy.

[11] Alanazi M, Mahoor M, Khodaei A (2020). Co-optimization generation and transmission planning for maximizing large-scale solar PV integration. Int. J. Electr. Power Energy Syst..

[12] Su Y, Chan L-C, Shu L, Tsui K-L (2012). Real-time prediction models for output power and efficiency of grid-connected solar photovoltaic systems. Appl. Energy.

[13] Aourir M, Abouloifa A, Lachkar I, Aouadi C, Giri F, Guerrero JM (2020). Nonlinear control and stability analysis of single stage grid-connected photovoltaic systems. Int. J. Electr. Power Energy Syst..

[14] Almeida D, Abeysinghe S, Ekanayake MP, Godaliyadda RI, Ekanayake J, Pasupuleti J (2020). Generalized approach to assess and characterise the impact of solar PV on LV networks. Int. J. Electr. Power Energy Syst..

[15] Wang Q, Yao W, Fang J, Ai X, Wen J, Yang X (2020). Dynamic modeling and small signal stability analysis of distributed photovoltaic grid-connected system with large scale of panel level DC optimizers. Appl. Energy.

[16] Wang G, Xin H, Wu D, Ju P (2019). Data-driven probabilistic small signal stability analysis for grid-connected PV systems. Int. J. Electr. Power Energy Syst..

[17] Munkhchuluun E, Meegahapola L, Vahidnia A (2020). Long-term voltage stability with large-scale solar-photovoltaic (PV) generation. Int. J. Electr. Power Energy Syst..

[18] Adewuyi OB, Lotfy ME, Akinloye BO, Rashid Howlader HO, Senjyu T, Narayanan K (2019). Security-constrained optimal utility-scale solar PV investment planning for weak grids: Short reviews and techno-economic analysis. Appl. Energy.

[19] Morjaria M, Anichkov D, Chadliev V, Soni S (2014). A grid-friendly plant: The role of utility-scale photovoltaic plants in grid stability and reliability. IEEE Power Energy Mag..

[20] Eftekharnejad S, Vittal V, Heydt GT, Keel B, Loehr J (2013). Small signal stability assessment of power systems with increased penetration of photovoltaic generation: A case study. IEEE Trans. Sustain. Energy.

[21] Refaat SS, Abu-Rub H, Sanfilippo AP, Mohamed A (2018). Impact of grid-tied large-scale photovoltaic system on dynamic voltage stability of electric power grids. IET Renew. Power Gener..

[22] Mensah LD, Yamoah JO, Adaramola MS (2019). Performance evaluation of a utility-scale grid-tied solar photovoltaic (PV) installation in Ghana. Energy Sustain. Dev..

[23] de Lima LC, de Araújo Ferreira L, de Lima Morais FHB (2017). Performance analysis of a grid connected photovoltaic system in northeastern Brazil. Energy Sustain. Dev..

[24] Komiyama R, Fujii Y (2019). Optimal integration assessment of solar PV in Japan’s electric power grid. Renew. Energy.

[25] Aziz AS, Tajuddin MFN, Adzman MR, Mohammed MF, Ramli MAM (2020). Feasibility analysis of grid-connected and islanded operation of a solar PV microgrid system: A case study of Iraq. Energy.

[26] Pietruszko SM, Gradzki M (2003). Performance of a grid connected small PV system in Poland. Appl. Energy.

[27] Zou H, Du H, Brown MA, Mao G (2017). Large-scale PV power generation in China: A grid parity and techno-economic analysis. Energy.

[28] Boddapati V, Daniel SA (2020). Performance analysis and investigations of grid-connected Solar Power Park in Kurnool, South India. Energy Sustain. Dev..

[29] Thotakura S, Chandan Kondamudi S, Xavier JF, Quanjin M, Reddy GR, Gangwar P (2020). Operational performance of megawatt-scale grid integrated rooftop solar PV system in tropical wet and dry climates of India. Case Stud. Therm. Eng..

[30] Bhattacharyya SC, Palit D, Sarangi GK, Srivastava V, Sharma P (2019). Solar PV mini-grids versus large-scale embedded PV generation: A case study of Uttar Pradesh (India). Energy Policy.

[31] Kumar M, Chandel SS, Kumar A (2020). Performance analysis of a 10 MWp utility scale grid-connected canal-top photovoltaic power plant under Indian climatic conditions. Energy.

[32] Malvoni M, Kumar NM, Chopra SS, Hatziargyriou N (2020). Performance and degradation assessment of large-scale grid-connected solar photovoltaic power plant in tropical semi-arid environment of India. Sol. Energy.

[33] Rose A, Stoner R, Pérez-Arriaga I (2016). Prospects for grid-connected solar PV in Kenya: A systems approach. Appl. Energy.

[34] Li DHW, Cheung KL, Lam TNT, Chan WWH (2012). A study of grid-connected photovoltaic (PV) system in Hong Kong. Appl. Energy.

[35] Sabo ML, Mariun N, Hizam H, Mohd Radzi MA, Zakaria A (2017). Spatial matching of large-scale grid-connected photovoltaic power generation with utility demand in Peninsular Malaysia. Appl. Energy.

[36] Papageorgiou A, Ashok A, Hashemi Farzad T, Sundberg C (2020). Climate change impact of integrating a solar microgrid system into the Swedish electricity grid. Appl. Energy.

[37] Sultan HM, Diab AAZ, Kuznetsov ON, Ali ZM, Abdalla O (2019). Evaluation of the impact of high penetration levels of PV power plants on the capacity, frequency and voltage stability of Egypt’s unified grid. Energies.

[38] Feilat EA, Azzam S, Al-Salaymeh A (2018). Impact of large PV and wind power plants on voltage and frequency stability of Jordan’s national grid. Sustain. Cities Soc..

[39] Tamimi B, Cañizares C, Bhattacharya K (2013). System stability impact of large-scale and distributed solar photovoltaic generation: The case of Ontario, Canada. IEEE Trans. Sustain. Energy.

[40] Esteban M, Portugal-Pereira J, McLellan BC, Bricker J, Farzaneh H, Djalilova N (2018). 100% renewable energy system in Japan: Smoothening and ancillary services. Appl. Energy.

[41] Yan R, Saha TK, Modi N, Masood N-A, Mosadeghy M (2015). The combined effects of high penetration of wind and PV on power system frequency response. Appl. Energy.

[42] Johnson SC, Rhodes JD, Webber ME (2020). Understanding the impact of non-synchronous wind and solar generation on grid stability and identifying mitigation pathways. Appl. Energy.

[43] Abdullahi D, Suresh S, Renukappa S, Oloke D (2017). Key barriers to the implementation of solar energy in Nigeria: A critical analysis. IOP Conf. Ser. Earth Environ. Sci..

[44] Eni RO, Akinbami JK (2017). Flexibility evaluation of integrating solar power into the Nigerian electricity grid. IET Renew. Power Gener..

[45] Adaramola MS (2014). Viability of grid-connected solar PV energy system in Jos, Nigeria. Int. J. Electr. Power Energy Syst..

[46] Adewuyi OB, Shigenobu R, Senjyu T, Lotfy ME, Howlader AM (2019). Multiobjective mix generation planning considering utility-scale solar PV system and voltage stability: Nigerian case study. Electr. Power Syst. Res..

[47] Adaramola MS, Paul SS, Oyewola OM (2014). Assessment of decentralized hybrid PV solar-diesel power system for applications in Northern part of Nigeria. Energy Sustain. Dev..

[48] Adeoye O, Spataru C (2018). Sustainable development of the West African Power Pool: Increasing solar energy integration and regional electricity trade. Energy Sustain. Dev..

[49] Owolabi AB, Nsafon BEK, Roh JW, Suh D, Huh J-S (2019). Validating the techno-economic and environmental sustainability of solar PV technology in Nigeria using RETScreen Experts to assess its viability. Sustain. Energy Technol. Assess..

[50] Adetokun BB, Ojo JO, Muriithi CM (2020). Reactive power-voltage-based voltage instability sensitivity indices for power grid with increasing renewable energy penetration. IEEE Access.

[51] Adetokun BB, Muriithi CM (2021). Impact of integrating large-scale DFIG-based wind energy conversion system on the voltage stability of weak national grids: A case study of the Nigerian power grid. Energy Rep..

[52] FICHTNER. *Transmission Expansion Plan Development of Power System Master Plan for the Transmission Company of Nigeria*. 8328P01/FICHT-19579512-v1 (2017).

[53] Nextier-Power. *Nigeria Electricity Market Intelligence Report_Q2* (2019).

[54] NERC. *Nigerian Electricity Regulatory Commission Quarterly Report: Second Quarter* (2019).

[55] Zhang HL, Baeyens J, Degrève J, Cacères G (2013). Concentrated solar power plants: Review and design methodology. Renew. Sustain. Energy Rev..

[56] Akwukwaegbu IO, Nosiri OC, Ezugwu EO (2017). Voltage stability investigation of the Nigeria 330KV interconnected grid system using eigenvalues method. American Journal of Engineering Research.

